# Animal Welfare and Economic Aspects of Using Nurse Sows in Swedish Pig Production

**DOI:** 10.3389/fvets.2017.00204

**Published:** 2017-12-01

**Authors:** Karin Alvåsen, Helena Hansson, Ulf Emanuelson, Rebecka Westin

**Affiliations:** ^1^Department of Clinical Sciences, Division of Ruminant Medicine and Epidemiology, Swedish University of Agricultural Sciences, Uppsala, Sweden; ^2^Department of Economics, Swedish University of Agricultural Sciences, Uppsala, Sweden; ^3^Gård och Djurhälsan, Skara, Sweden

**Keywords:** piglet, pig industry, modeling, contribution margin, stochastic simulation

## Abstract

The number of born piglets per litter has increased in Swedish pig industry, and farmers are struggling to improve piglet survival. A common practice is to make litters more equally sized by moving piglets from large litters to smaller to make sure that all piglets get an own teat to suckle. Litter equalization is not always enough, as many sows have large litters and/or damaged teats, which results in an insufficient number of available teats. One way to solve this problem is to use nurse sows. A nurse sow raises, and weans, her own piglets before receiving a foster litter. The objectives of this study were to address how the use of nurse sows affects the welfare of sows and piglets and to explore how it impacts the contribution margin of pig production in Sweden. A literature search was made to investigate welfare aspects on sows and piglets. As there were few published studies on nurse sows, an expert group meeting was organized. In order to explore the impact on the contribution margin of pig production, a partial budgeting approach with stochastic elements was used for a fictive pig farm. Standard templates for calculating costs and benefits were supplemented with figures from existing literature and the gathered expert opinions. In Sweden, the minimum suckling period is 28 days while published studies involving nurse sows, all from outside of Sweden, weaned the piglets at 21 days. A Swedish nurse sow will thus get longer lactation period which might increase the risk of poor body condition, damaged teats, and shoulder ulcers. This indicates a reduced welfare of the sow and may lead to impaired fertility and increased culling risk. On the other hand, the piglet mortality could be reduced with the use of nurse sows, but the separation and mixing of piglets could be stressful. The partial budgeting suggested that the nurse sow system is slightly more profitable (+6,838 Swedish krona) per farrowing group during one dry and one lactation period compared to the conventional system. The result is, however, highly dependent on the input values, and welfare aspects were not considered in the calculations.

## Introduction

Breeding programs toward hyperprolific sows have resulted in sows that produce a surplus of piglets compared to the number of functional teats (1). The Swedish national average of live-born pigs per litter has risen from 13.1 to 14.0 from 2011 to 2016 (2), and the average litter size is expected to rise even further. The intention with the increased litter size is a more efficient and profitable pig production by a higher number of piglets that can be weaned and later slaughtered, without increasing the number of sows. However, in large litters more piglets will have to share the resources (e.g., milk), and a sow usually has 14 teats and can thus feed up to 14 piglets. In today’s production, a sow can give birth to more than 20 live-born piglets. If no action is taken, not all of these piglets can be expected to survive.

Most often farrowing occurs batch wise, i.e., a whole group of sows farrows within a few couple of days. This makes it possible to move piglets from large litters to sows with smaller litters, known as “litter equalization” (1). In this manner, the survival rates might increase (3). If the number of functional teats in the farrowing group is not sufficient, surplus piglets can be gathered and placed with a nurse sow. A nurse sow is a lactating sow from another farrowing group that have just weaned her own litter (4). The nurse sow is moved into the group of fresh sows and will continue to be suckled by a new litter of foster piglets. Nurse sows can be used in one-step or two-step systems (1). A one-step system means that a sow, immediately after weaning her own piglets, is moved to a foster litter with newborn “surplus piglets”. The one-step nurse sow will have a total lactation period of at least 8 weeks (28 + 28 days) under Swedish conditions. Another way to use nurse sows is to do it in two steps. In this way two sows are needed. Sow 1 nurses her own litter for 4–8 days and then the whole litter is moved to Sow 2, which has just weaned her own litter. Sow 1 receives newborn surplus piglets and nurses them for at least 28 days if in Sweden. Sow 1 will have a total lactation period of approximately 5 weeks (4–8 + 28 days) which is comparable to the average lactation period in Sweden [33 days (2)]. Sow 2 will have a prolonged lactation period of approximately 3 weeks depending on how old the foster litter is when it is moved. If the foster litter is 4 days when transferred, the nurse sow needs to have them for at least 24 days before they can be weaned. This gives a total minimum lactation period of 7.4 weeks (28 + 24 days) for Sow 2 if she weaned her own litter after 28 days. The overall lactation period for the nurse sows is shorter in the two-step nurse system. Older foster piglets are also more easily accepted compared to newborn by sows at weaning (5). The two-step nurse system is known to be more commonly used in Denmark where nurse sows are used to a wide extent (6).

The Swedish legislation has more stringent rules regarding the keeping of sows and piglets during suckling than the European Council Directive (7). Sows in Sweden should be loose housed at both farrowing and suckling (SJVFS 2010:15), which is not a requirement in the rest of the EU where the sows can be kept in crates during this period (7). Furthermore, in Sweden, piglets have to be at least 28 days at weaning. This is generally valid throughout the EU, but an exception to the regulations makes it possible to wean piglets already at 21 days, which is widely practiced in nurse sow systems in order to limit the overall lactation period for the nurse sow (1). This means that nurse sows in Sweden will have longer lactation periods compared to nurse sows in other countries, and this may have an impact on their welfare, but the welfare aspects of nurse sow systems in Sweden have not been addressed so far.

There are also no published studies on the economic aspects of using nurse sows under Swedish production conditions. The objectives of this study were therefore to: (1) discuss possible animal welfare consequences associated with using nurse sows under Swedish conditions, with a standpoint from available published literature and expert opinions and (2) evaluate the effects of using nurse sows on the contribution margin of piglet production in Sweden.

## Materials and Methods

As there is a paucity of literature on nurse sows, and no published studies from Sweden, an expert group meeting was organized to gather information on the nurse sow system and its effects on animal welfare and production parameters. This information was a necessary first step to build an economic model as results and figures from available literature could not be directly transferred into the economic models as the Swedish production conditions differed in many ways. The expert group meeting was arranged in Uppsala, Sweden, in August 2016 and included persons from the industry working as herd health veterinarians, production advisors, and researchers. The meeting started with a brief presentation of the results from a literature review consisting of 36 references including published papers, scientific reports, and a bachelor thesis. Different animal welfare aspects of using nurse sows on herd level as well as individual level, both from the sow and piglet perspective, were then discussed according to a structured protocol. In the next step, the economic parameters included in a standard template (8) were discussed one by one. Finally, the group agreed collectively on which input variables to include in the economic model for this study as well as their effects. The expert group meeting lasted for 3 h.

To estimate the economic aspects of using nurse sows, a partial budget (contribution-margin)-based stochastic farm-level model was developed in Microsoft Excel 2013 (Microsoft Corp., Redmond, WA, USA). In this way, we could isolate the effects on the contribution margin of using nurse sows compared to a conventional system, by only focusing on the economic variables (revenue and costs) likely to depend on the system. The model was based on a standard template (8), but modified according to experts’ opinions to include most variables affected by using nurse sows. Input variables were based on Swedish pig production data from 2015 and 2016 (9) and the results from the expert group meeting. The input variables are listed in Tables 1 and 2.

**Table 1:** Overview of stochastic input variables used in the partial budgeting model.

Input variable	System	Mean (SD)	Mode; min; max	Distribution	Reference
Number of live-born piglets per litter	Both	13.7 (0.8)		Normal	(2)

Piglet mortality rate (deaths/100 piglet-years)	Conventional		0.18; 0.08; 0.33	Triangular	WinPig and expert opinion
	Nurse sow		0.14; 0.07; 0.25	Triangular	Expert opinion

Weight at sale (79 days)	Conventional	31 (3)		Normal	(2)
	Nurse sow	31 (2)		Normal	Expert opinion

Feed consumption during lactation (MJ per week)	Both		510; 490; 530	Triangular	TN-70 feed recommendation, 2016

Feed consumption during dry period (MJ per week)	Both		220.5; 245; 269.5	Triangular	TN-70 feed recommendation, 2016

**Table 2:** Overview of deterministic input variables used in the partial budget model of economic consequences of using nurse sows.

Input variable	Fixed	Reference
Price at sale [Swedish krona (SEK)/79 days old piglet]	580	HK Scan, 2016
Additional bonus at sale if piglet batch weight > 30 kg (SEK/extra kg)	6	HK Scan, 2016
Feed consumption piglet (kg/week)	1	Expert opinion
Price of feed during lactation (SEK/MJ)	0.22	(10)
Price of feed during dry period (SEK/MJ)	0.20	(10)
Price of piglet feed (SEK/kg)	6	
Semen costs (SEK/unit)	40	(11)

The model scenario was based on a farm with three farrowing stalls and farrowings fortnightly. The conventional system had 590 sows/year, and the nurse sow scenario had 567 sows/year. Each farrowing group was kept in stalls with 50 farrowing pens. In the conventional system 50 pregnant sows were housed in the pens at start, while in the nurse sow scenario 48 pregnant sows were housed and the remaining 2 pens were used for surplus piglets, and two-step nurse sows (from another farrowing group) were later moved to these pens. The sow groups were moved to the farrowing unit 4 days before expected farrowing and kept in that unit until all piglets had reached 28 days of age. In this way, the sow group stayed in the farrowing unit for a 5-week period.

The basic model was deterministic, however, some key variables were modeled stochastically (Tables 1 and 2). A stochastic model takes the parameter variation into calculation to generate results with a distribution, representing uncertainty in results (12). The stochastic elements of the model were handled with @RISK 7.5 (Palisade Corp., Ithaca, NY, USA), an Excel add in, which performs risk analysis using Monte Carlo simulations. In each simulation, 5,000 iterations and Latin Hypercube sampling were used with a static seed of 31,517 to ensure that all simulations provided repeatable results.

The output variables affecting the contribution margin in the partial budgeting were “Revenue from sold piglets,” “Feed costs”, and “Semen costs.” “Revenue from sold piglets” was calculated by using the number of weaned piglets per farrowing group (the number of live-born piglets per farrowing group × piglet mortality rate) and the price at sale [580 Swedish krona (SEK) per 30 kg batch-pig weight with an addition of 6.5 SEK per kg for batches with average weights > 30.1 kg/pig]. “Feed costs” consisted of the number of feed weeks × feed consumption × feed price (for all categories, i.e., sows in dry period, lactating sows, and growing piglets). “Semen cost” was calculated as the number of sows × cost for semen. A correlation between number of live-born piglets and piglet mortality rate for the conventional system and the nurse sow system was set to 0.8 and 0.5, respectively. The impact on contribution margin (revenues minus costs) of using nurse sows was compared to a conventional situation and was calculated at batch level for one farrowing group (50 pens) from insemination (included one dry and one lactation period). The full economic model is shown in Table S1 in Supplementary Material.

Further contact was made with the expert group to sort out upcoming queries and to validate the model. Finally, a sensitivity analysis was used to evaluate input variables with strong impact on the model outcome. This was conducted using the sensitivity analysis function of @Risk. Regression tornado diagrams, in which @Risk runs a multiple regression analysis for each iteration with the outcome of interest and the simulated (standardized) values of the stochastic variables as independent variables, were carried out. This analysis shows the mean of the 10% lowest and highest simulated values (Tornado graph “change in output mean”) and the change in the outcome variable when the independent variables increase by 1 SD with all other variables being constant (Tornado graph “regression mapped values”).

## Results

### Welfare and Production Aspects

During the expert group meeting, the participants concluded that consequences of being a nurse sow will be highly dependent on the farmers’ skills in selecting appropriate individuals. Parity, lactation stage, maternity traits, and robustness of the nurse sow will be of major importance.

An early separation will most likely cause negative stress, both for the sows and for the piglets (13). In the first few days after farrowing, the teat order is established as each piglet has a particular pair of teats to suckle (14). In litter equalization, foster piglets are mixed with the sow’s biological piglets, and this might start a fight over the teats. In the case of late litter equalization, the suckling is affected negatively, as the fighting piglets make the sow restless, which results in more disrupted nursings and deficient milk ejection (15). In the two-step nurse sow system, all the sows’ biological piglets are removed. In this way, the teat competition might be less severe as all in the new litter are foster piglets (5). In a recent study by Amdi et al. (16), no differences in suckling frequency between nurse and non-nurse sows could be detected. This indicates that being a foster piglet in the nurse sow litter might be similar or less stressful than being exposed to litter equalization which is a common practice in Sweden.

Several studies have shown that it might take up to 12 h before the nurse sow accepts the new litter and allows the foster piglets to suckle (5, 17, 18). Generally, two-step nurse sows are considered to accept the foster piglets quicker compared to nurse sows in the one-step system (5). It can take over 12 h before the sow allows the foster piglets to suckle and in some cases the nurse sow never accepts the foster litter (19). Thorup and Sørensen (5) compared nurse sows in the one-step and two-step systems, and seven of eight sows let the foster piglets to suckle within 12 h if they had nursed their own litter for 1 week and then received newborns (Sow 1 in two-step systems). Of the sows that received newborns after 3 weeks of nursing her own litter (one-step system), only three of eight sows allowed the foster litter to suckle within 12 h. This period of starvation affected the piglet mortality in the foster litters. Mortality was 6% in sows allowing them to suckle within 12 h compared to 20% in litters where it took over 12 h. However, the risk of starvation (and eventually death) is high also in the conventional system if the litter size exceeds the number of functional teats. In cases where the sow accepts the foster litter, mortality risks in foster litters are not higher than those seen in conventional litters. Bruun et al. (20) found that non-nurse sows on average weaned 11.65 piglets/litter, while nurse sows weaned 12.41 piglets in their own litter and 11.48 piglets in the foster litter. Moreover, the animal welfare-related consequences for piglets in nurse sow systems are assumed to be similar to what have been reported from other countries, as the moving of piglets occurs at the same time after birth regardless of the total length of the suckling period.

The majority of the nurse sow studies have been carried out in countries where sows are kept in farrowing crates throughout the suckling period. Since a loose-housed sow has a greater ability to move around, and thus more easily can avoid the foster piglets, it is possible that it takes even longer time before loose-housed sows nurse the foster litter. This imposes greater challenges for the foster piglets in nurse sow systems in Sweden. In a Swedish study by Nilsson and Larsson (21), only 2 of 18 nurse sows (11%) allowed the foster litter to suckle within 6 h. Twelve nurse sows (67%) allowed them to suckle after 6–12 h, and four sows (22%) allowed the foster piglets to suckle after 12 h. This time of starvation will have a negative effect on the piglets and might cause death in weak piglets.

Furthermore, an extended lactation period may deplete the sows’ body lipid and protein reserves and cause damaged teats. A low body condition score increases the risk of shoulder wounds (22) and impaired fertility (23). A cross-sectional study conducted in 57 sow herds in Denmark showed that nurse sows had significantly higher prevalence of udder wounds and swollen bursa on legs compared to conventional sows (6). There were, however, no differences in body condition or prevalence of shoulder ulcers. This could be due to that farmers scored body condition as one of the most important factors when choosing a nurse sow. As the overall lactation period for a Swedish nurse sows can be as long as 8 weeks, due to our strict animal welfare regulation regarding weaning age, loss of body condition is very likely to occur. Selecting sows with a good body condition score, therefore, seems to be equally or even more important under Swedish conditions.

Several studies have shown that the interval between weaning and conception is longer for nurse sows (20, 21, 24). In a study by Bruun et al. (20), based on data from nearly 80,000 litters, the nurse sows (defined as Sow 2 in a two-step system) had 4.23 days between weaning to conception compared to the 4.19 days found in conventional sows. The nurse sows in that study had an average lactation length of 40.3 days versus 27.8 days for the conventional sows. There was, however, no difference in the rate of sows returning to estrus.

The temporary prolonged nursing interval that arises when separating the sow from her own litter and before accepting the new foster litter has been shown to be sufficient to induce heat in some individuals (24). In these cases, the sow gets unsynchronized with the rest of the farrowing group, which can cause problems in the batch-wise production. Furthermore, loss of body condition in sows and a long period of starving before the nurse sow accepts the foster litter and allows piglets to suckle were considered as the main risk factors for reduced welfare under Swedish conditions.

### Economic Aspects

The economic model demonstrated that the contribution margin was slightly improved in the scenario with nurse sows compared to the conventional situation. The mean contribution margin in the conventional system and the nurse sow system was 232,448 SEK (SD = 16,586) and 239,286 SEK (SD = 23,733), respectively, for one farrowing group with 50 available pens followed during one lactation and dry period. The differences between the systems ranged from −74,880 to 95,552 SEK with the mean value of 6,838 SEK (SD = 27,773). The nurse sow system was beneficial compared to the conventional system in 58% of the iterations.

**Figure 1:**
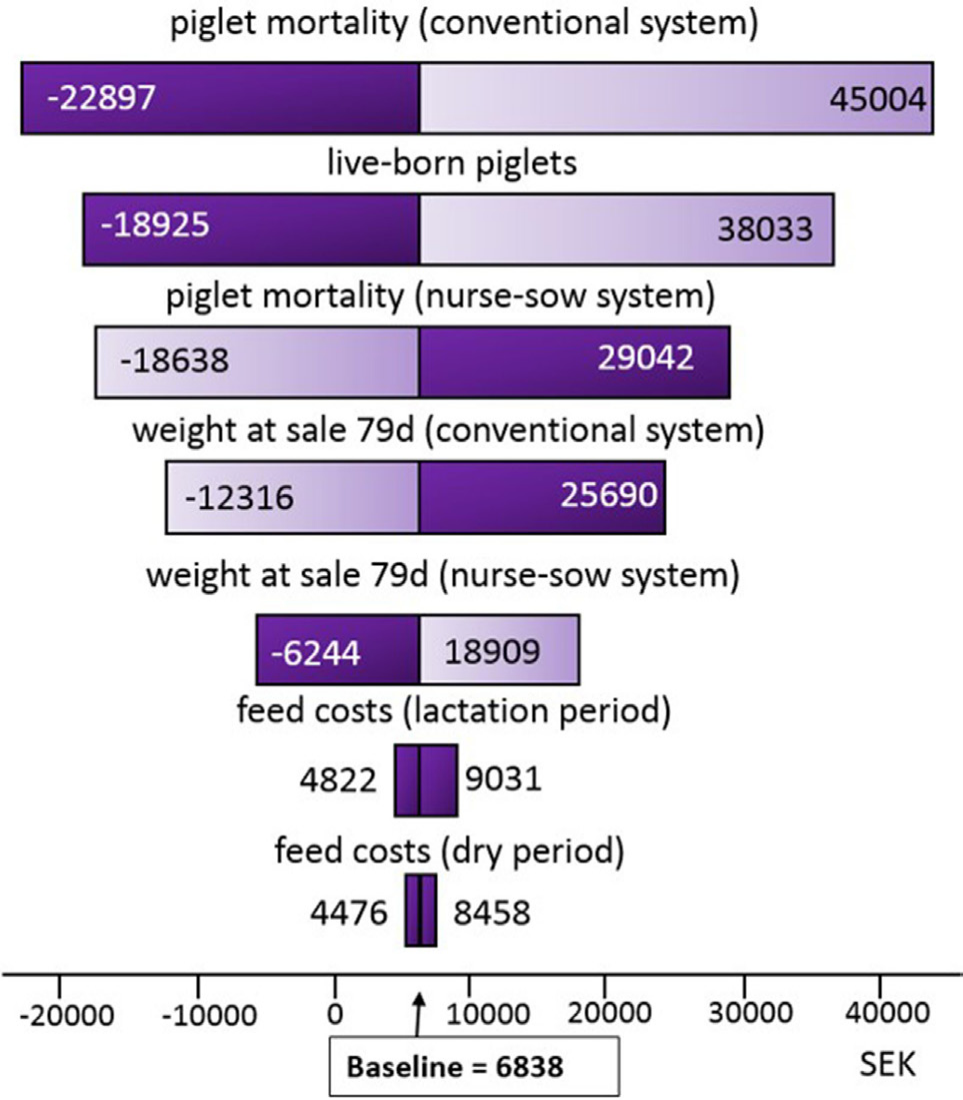
The input variables with greatest impact on the partial budget analysis comparing contribution margin of a nurse sow system with a conventional system. Values on either side of the bar represent the mean of the 10% lowest and 10% highest simulated values in Swedish krona for each variable.

**Figure 2:**
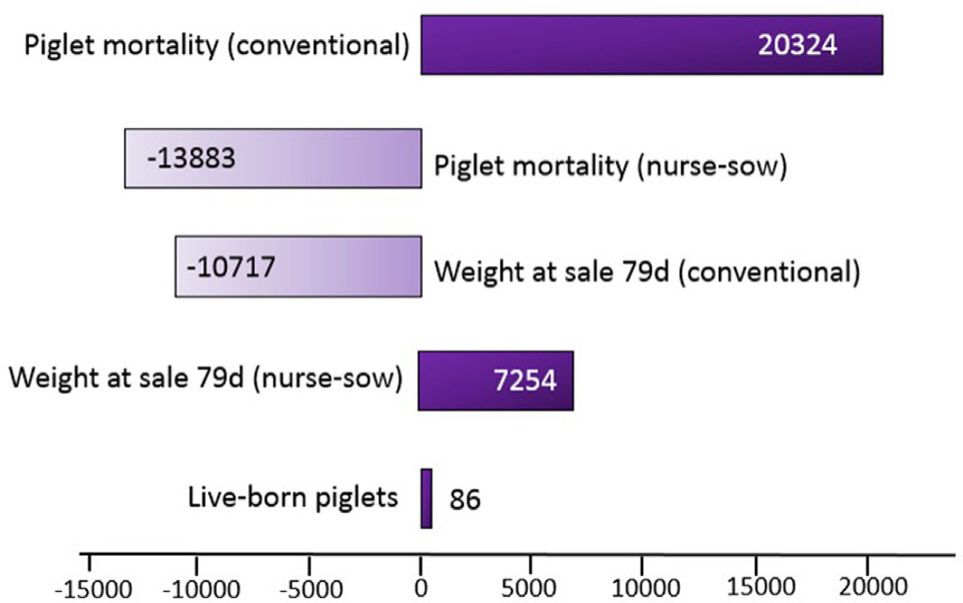
A tornado graph (with regressed mapped values) demonstrating the change in contribution margin (Swedish krona) if the input variables are increased by 1 SD and the other variables are held constant.

In Figure 1, the sensitivity analysis tornado diagram shows that piglet mortality, number of live-born piglets, and weight at sale had the greatest impact on the results. The other inputs in the model had minimal impact. The tornado graph in Figure 2 demonstrated that piglet mortality and weight at sale had greatest impact on the contribution margin when the independent variables were increased by 1 SD. For instance, the benefit of using nurse sow system increased as the piglet mortality rate in the conventional system increased by 1 SD, and the opposite scenario was seen when piglet mortality rate increased in the nurse sow system. Changing the price at sale with +10% resulted in a higher contribution margin (7,308 SEK) and reducing the price at sale with −10% reduced the contribution margin (6,368 SEK) slightly. The nurse sow system was, however, favorable in both scenarios.

## Discussion

The objectives of this study were to discuss the animal welfare aspects and to explore the economic aspects of using nurse sows in Swedish pig production. The results suggest that there are animal welfare concerns both for sows and piglets that need to be considered and also that using nurse sows can provide a slightly better economic performance than conventional practice on Swedish farms. However, it should be noted that in the economic model, the definition of contribution margin did not include cost items related to salary and veterinary costs, as these variables are considered relatively constant in the short run. Veterinary costs at pig farms in Sweden are usually constant because almost all farms have a fixed number of scheduled veterinary visits per year and need then not be included in a contribution margin analysis (which is conducted within the limits of given fixed resources). Salary was not added to the model as the amount of person-hours needed in the analysis was too difficult to estimate, because only a bachelor thesis on labor use in nurse sow systems in Sweden has been done. Moreover, labor is a production factor that in reality can be considered fixed in the short run, as it usually takes time to recruit new employees and it could be difficult finding persons interested in part-time employment, and should then by definition not be included in the calculation of contribution margin. Furthermore, the animal welfare implications are not included in the economic model, even though there are several important animal welfare aspects regarding both sows and piglets. Direct economic effects of such aspects are, however, difficult to assess and research into ways to transform ethical values into monetary terms is strongly advocated.

The general goal with nurse sows is to increase the piglet survival rate (from birth to weaning). In our scenario, the mortality rate was set to be higher in the conventional system, an input which was based on the expert solicitation exercise because no studies were available. The sensitivity analysis showed that this variable had the largest influence on the outcome, and it would therefore be important to estimate piglet mortality in nurse sow systems, especially under Swedish conditions, to verify our results. The second most influential variable was number of live-born piglets, but the input value of variable was based on large number of production records, and the sensitivity analysis thus shows the effect of an inherent variability.

It is important to remember that there is a large variability in most input variables on commercial farms, and the results from this study only give an indication that the nurse sow system can be profitable under average Swedish production conditions. The beneficial potential of using nurse sows will highly depend on the conditions and management routines at specific farms. The economic model in this study included only a few number of variables. Parameters not included in this economic model, e.g., labor, may influence the results, and a cautious interpretation of the results is recommended. There could also be other important welfare aspects (e.g., behavioral and physiological parameters), additional to the ones discussed in this paper, which need to be considered in future studies.

## Conclusion

Using nurse sows is one way to reduce piglet mortality rates from birth to weaning. The Swedish national average of live-born piglets is 14.0 per litter. Breeding toward larger litter sizes will result in lower average birth weights for piglets, but also to an increased risk of non-sufficient colostrum intake because of the competition for functioning teats. This study explored the animal welfare and economic consequences of using nurse sows under Swedish production conditions to overcome some of these problems. In the nurse sow system (assuming a fixed number of farrowing pens), there will be less sow-years compared to the conventional situation. The partial budget analysis showed a higher contribution margin for the nurse sow system. Animal welfare aspects were, however, not included in the economic model, and due to the limited number of input variables the results should be interpreted with caution. There are important animal welfare concerns that need to be studied further, especially under Swedish production conditions.

## Author Contributions

All authors contributed to the design of the work. KA, HH, and RW participated in the expert group meeting. KA, HH, and UE performed the analysis. KA drafted the manuscript, and all the authors read and approved the final manuscript.

## Conflict of Interest Statement

The authors declare that the research was conducted in the absence of any commercial or financial relationships that could be construed as a potential conflict of interest.
