# Supplementary material for: Animal Welfare and Economic Aspects of Using Nurse Sows in Swedish Pig Production
Source: Front Vet Sci. 2017 Dec 1;4:204. doi: 10.3389/fvets.2017.00204 (PMC5770636; doi:10.3389/fvets.2017.00204)
Supplement: Supplementary file 1 [file Table_1.DOCX]

Supplementary Material

Animal welfare and economic aspects of using nurse sows
in Swedish pig production

Alvåsen K*, Hansson H, Emanuelson U, Westin R

*** Correspondence:** Corresponding Author: karin.alvasen@slu.se

## Supplementary Table

**Supplementary Table 1.** The economic model used to calculate contribution margin of the conventional system (without nurse sows) and the nurse sow system (where two nurse sows are used). A yellow box indicates a deterministic input value and a blue box indicates a stochastic input value. Uncolored boxes are calculated in the way that is explained in the last column. The presented figures are an example from one random simulation.

| **Input variable** | | **Conventional** | | **Nurse sow** | | **Explanation** | |  | |
| --- | --- | --- | --- | --- | --- | --- | --- | --- | --- |
|  |  |  |  |  |  |  |  |  | |
| Sows (n) |  | 50 |  | 48 |  |  |  |  | |
| Live-born piglets/sow (n) | |  | 13,7 |  |  |  |  |  | |
| Live-born piglets/farrowing group (n) | | 685 |  | 658 |  | "Sows" x "Live-born piglets per sow" | | |  |
|  |  |  |  |  |  |  |  |  | |
| Piglet mortality risk (%) | | 0,2 |  | 0,14 |  |  |  |  | |
| Weaned piglets/farrowing group (n) | | 550 |  | 566 |  | "Live-born piglets/farrowing group" x (1- "piglet mortality risk") | | |  |
| Weight at sale 79 d (kg/piglet) | | 31,6 |  | 31,6 |  |  |  |  | |
|  |  |  |  |  |  |  |  |  | |
| Price at sale (SEK/30kg piglet batch weight) | |  | 580 |  |  |  |  |  | |
| Additional bonus at sale if batch  weight >30kg (SEK/extra kg) | | | 6,5 |  |  |  |  |  | |
| Revenue per sold 79 d piglet (SEK) | | 589,1 |  | 589,8 |  | "Price at sale" + ("Weight at sale 79 d" - 30) x "Additional bonus at sale if batch weight > 30 kg" | | |  |
|  |  |  |  |  |  |  |  |  | |
| Feed weeks: Lactating sows | | 250 |  | 240 |  | "Sows" x 5 weeks | |  | |
| Feed weeks: Dry sows | | 825 |  | 792 |  | "Sows" x 16,5 weeks | | |  |
| Feed weeks: Nurse sows (Sow 1 in Figure 2) | |  |  | 2 |  | Sow 1 gets 4-7 days extra lactation period (1 week x 2 sows) | | |  |
| Feed weeks: Nurse sows (Sow 2 in Figure 2) | |  |  | 7 |  | Sow 2 gets one extra lactation period of 21-24 days (3,5 weeks x 2 sows) | | |  |
|  |  |  |  |  |  |  |  |  | |
| Feed requirement (MJ)/week: lactation | |  | 510 |  |  |  |  |  | |
| Feed requirement (MJ)/week: dry period | |  | 245 |  |  |  |  |  | |
| Feed requirement: piglet | | 3088 |  | 3058 |  | (("Live-born piglets/farrowing group" x "Weaned piglets/farrowing group") / 2) x 5 weeks | | |  |
| Price of feed: lactation (SEK/MJ) | |  | 0,22 |  |  |  |  |  | |
| Price of feed: Dry period (SEK/MJ) | |  | 0,20 |  |  |  |  |  | |
|  |  |  |  |  |  |  |  |  | |
| Cost of feed: Lactating sows | | 28050 |  | 27938 |  | “Feed weeks: Lactating sows" x "Feed requirement (MJ)/week: lactation" x "Price of feed: lactation" | | |  |
| Cost of feed: Dry sows | | 40425 |  | 38808 |  | Feed weeks: dry period x "Feed requirement (MJ)/week: dry period" x "Price of feed: Dry Sows" | | |  |
| Cost of feed: piglets | | 184530 |  | 18347 |  | "Feed requirement: piglet" x 6 SEK/kg | | |  |
| Insemination (SEK/dose) | |  | 40 |  |  |  |  |  | |
|  |  |  |  |  |  |  |  |  | |
|  |  |  |  |  |  |  |  |  | |
| **Calculations Partial Budget** | |  |  |  |  |  |  |  | |
|  |  |  |  |  |  |  |  |  | |
| Returns piglet sale | | 324172 |  | 333525 |  | "Weaned piglets/farrowing group" x "Revenue per sold 79 d piglet" | | |  |
|  |  |  |  |  |  |  |  |  | |
| Feed cost |  | 87004 |  | 85093 |  | "Cost of feed: Lactating sows" + "Cost of feed: Dry sows" + "Cost of feed: piglets" | | |  |
| Cost of insemination | | 2000 |  | 1920 |  | "Sows" x "Feed cost" | | |  |
|  |  |  |  |  |  |  |  |  | |
| Contribution margin | | 235168 |  | 246512 |  | "Returns piglet sale" - ("Feed cost" - "Cost of insemination") | | |  |
|  |  |  |  |  |  |  |  |  | |
| **Result** |  |  | 11344 | SEK |  | "Contribution margin nurse sow system" - "Contribution margin conventional system" | | |  |
|  | | | | |  |  |  |  | |
|  |  |  |  |  |  |  |  |  | |
